# Microfluidic Platforms to Unravel Mysteries of Alzheimer’s Disease: How Far Have We Come?

**DOI:** 10.3390/life11101022

**Published:** 2021-09-28

**Authors:** Pragya Prasanna, Shweta Rathee, Vedanabhatla Rahul, Debabrata Mandal, Macherla Sharath Chandra Goud, Pardeep Yadav, Susan Hawthorne, Ankur Sharma, Piyush Kumar Gupta, Shreesh Ojha, Niraj Kumar Jha, Chiara Villa, Saurabh Kumar Jha

**Affiliations:** 1 School of Applied Sciences, KK University, Nalanda 803115, Bihar, India; mscgoud1234@gmail.com; 2 Department of Food Science and Technology, National Institute of Food Technology, Entrepreneurship and Management, Sonipat 131028, Haryana, India; shweta.r@niftem.ac.in; 3 Department of Mechanical Engineering, National Institute of Technology, Rourkela 769008, Odisha, India; vrahul1803@gmail.com; 4 Department of Biotechnology, National Institute of Pharmaceutical Education and Research, Hajipur 844101, Bihar, India; debabrataman@gmail.com; 5 Department of Biotechnology, School of Engineering and Technology (SET), Sharda University, Greater Noida 201310, Uttar Pradesh, India; par.yadav2011@gmail.com (P.Y.); niraj.jha@sharda.ac.in (N.K.J.); 6 School of Pharmacy and Pharmaceutical Sciences, Ulster University, Cromore Road, Coleraine, Co., Londonderry BT52 1SA, UK; s.hawthorne@ulster.ac.uk; 7 Department of Life Sciences, School of Basic Science and Research (SBSR), Sharda University, Greater Noida 201310, Uttar Pradesh, India; ankur.sharma7@sharda.ac.in (A.S.); dr.piyushkgupta@gmail.com (P.K.G.); 8 Department of Pharmacology and Therapeutics, College of Medicine and Health Sciences, P.O. Box 17666, United Arab Emirates University, Al Ain 15551, United Arab Emirates; shreeshojha@uaeu.ac.ae; 9 School of Medicine and Surgery, University of Milano-Bicocca, 20900 Monza, Italy; chiara.villa@unimib.it

**Keywords:** Alzheimer’s disease, microfluidics, lab-on-chip, 3D culture, organ-on-chip

## Abstract

Alzheimer’s disease (AD) is a significant health concern with enormous social and economic impact globally. The gradual deterioration of cognitive functions and irreversible neuronal losses are primary features of the disease. Even after decades of research, most therapeutic options are merely symptomatic, and drugs in clinical practice present numerous side effects. Lack of effective diagnostic techniques prevents the early prognosis of disease, resulting in a gradual deterioration in the quality of life. Furthermore, the mechanism of cognitive impairment and AD pathophysiology is poorly understood. Microfluidics exploits different microscale properties of fluids to mimic environments on microfluidic chip-like devices. These miniature multichambered devices can be used to grow cells and 3D tissues in vitro, analyze cell-to-cell communication, decipher the roles of neural cells such as microglia, and gain insights into AD pathophysiology. This review focuses on the applications and impact of microfluidics on AD research. We discuss the technical challenges and possible solutions provided by this new cutting-edge technique to understand disease-associated pathways and mechanisms.

## 1. Introduction

Alzheimer’s disease (AD) is a chronic neurodegenerative condition in which cognition and memory formation decline progressively due to an irreversible loss of neurons in the hippocampus and cortex regions [1]. It is characterized by the extracellular formation of senile plaque mainly constituted by amyloid-beta 42 (Aβ42)peptide and intracellular neurofibrillary tangles (NFTs), composed of hyper-phosphorylated paired helical filaments of the microtubule-associated protein tau (MAPT) [2,3,4]. Apart from Aβ and tau pathology, processes such as impaired synaptic functions, neurotransmission dysfunction, and microglia-mediated inflammation play a key role in AD pathogenesis [5]. Primary symptoms of the disease comprise memory deterioration, apathy, depression, and changes in personality and behavior that finally require full-time medical care [6]. The majority of AD cases present as a late-onset sporadic form (SAD) occurring in individuals aged 65 or older. SAD shows a complex etiology and results from a combination of genetic and environmental influences. To date, the only confirmed genetic risk is represented by the presence of the ε4 allele of Apolipoprotein E (ApoE), the main carrier of cholesterol in the central nervous system (CNS). This variant accelerates the onset of AD by enhancing the Aβ deposition into plaques and reducing its clearance from the cerebral tissue [7]. On the contrary, the rare early-onset forms of AD are familiar with FAD with an autosomal dominant pattern of inheritance in one of the known genes, *APP*, *PSEN1*, and *PSEN2*, encoding the Aβ precursor protein (APP), presenilin-1, and presenilin-2, respectively. As all of these are involved in the maturation and processing of APP, mutations in these genes result in increased production or aggregation of Aβ peptides [8]. The ‘World Alzheimer Report 2019’ shows that AD accounts for more than 70% of the total dementia cases diagnosed worldwide [9,10], therefore an early diagnosis of AD is crucial for disease management [11].

Despite AD prevalence and many years of research, several aspects of its complex etiology remain unexplored [12,13]. Moreover, the current therapeutic strategies are merely symptomatic, attenuating only behavioral symptoms but presenting several side-effects such as confusion, dizziness, depression, constipation, and diarrhea, reported in most medications [14]. Therefore, a more in-depth understanding of the molecular mechanisms underlying AD pathogenesis, revisiting numerous existing concepts, and effective screening for therapies aimed at halting or preventing neurodegeneration in AD is required [15,16]. The lack of suitable experimental models has also presented a bottleneck in understanding the AD pathological mechanism. Moreover, widely accepted notions such as the deposition of Aβ and hyperphosphorylation of microtubular protein tau also lack a direct correlation between the deposition or phosphorylation and the disease progression [17,18].

In recent years, microfluidics is emerging as an economical and versatile platform for biologists to mimic and control the cellular microenvironment in order to model diseases, study cell behavior from single- to multi-cellular organism level, and develop multiple experiments in miniaturized devices suitable for diagnostics, biomedical analysis, pathological studies of neural degeneration and drug developments [19,20]. These devices are popular, especially for their flexibility of design, experimental flexibility, leverage of a sufficient number of controls, handling single cells, controlled co-culture, reduced reagent consumption, reduced contamination risk, and efficient high throughput experimentation.

The past decade has witnessed a surge in the use of microfluidics technology in neurodegenerative diseases to gradually minimize biomedical research dependence on in vivo models [21]. These platforms have been widely implicated in growing 3D gels that could be further applied in producing a three-dimensional tissue representative of human organs. With the help of these miniaturized devices, the growth of neurons, astrocytes, and microglia have also been facilitated in the form of triculture models [22]. This review describes the latest advances in the progress of microfluidics technologies and elaborates various ways through which the domain of microfluidics presents solutions to the management of neurodegenerative disease, with a particular focus on AD. First, we emphasized the applications of microfluidics in the study of disease pathophysiology and the early detection of AD with the help of known biomarkers at a miniaturized level. Subsequently, we examined the impact of microfluidics on accelerating AD research. We then discussed the possible challenges that this field needs to overcome and the directions to be taken before realizing its full-fledged application in the AD field.

## 2. Revisiting Alzheimer’s Disease: What Is Known?

### 2.1. History

AD was first diagnosed by a German psychiatrist and neuropathologist, Alois Alzheimer, in 1906 [16]. However, after 1907, the behavioral and physiological changes in AD and naturally occurring senility and dementia were differentiated [23]. Symptoms such as failure of storage and retrieval of memory, confusion, and poor judgment have been categorized as characteristic features of AD. Other symptoms include language disturbance, agitation, withdrawal, and hallucinations followed by occasional seizures, increased muscle tone, and mutism [1,9,10,12]. Even after decades of research, the social and economic impact of the disease has not decreased, and the projections of the World Alzheimer Report 2019 predict more than 150 million cases by 2050 [9,10,12]. Due to remarkable advances in science and technology, increased understanding of the disease pathophysiology and causes has led to improved diagnosis and treatment [13,24].

### 2.2. Causes

**Figure 1:**
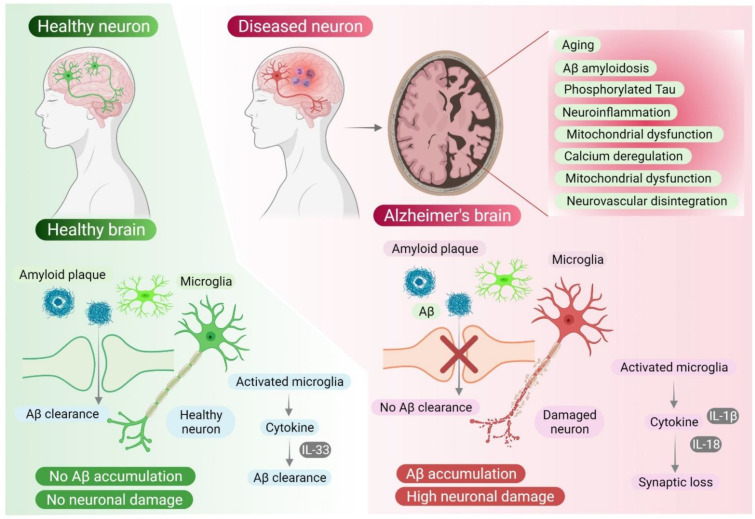
The pathophysiology of Alzheimer’s disease is very complex. Major pathological hallmarks of Alzheimer’s disease are provided. Among all the hallmarks, Aβ accumulation is considered the major cause of neurodegeneration in Alzheimer’s disease. It has been found that all other causes such as tau pathology and/or neuroinflammation ultimately converge to Aβ accumulation. For instance, microglia, the innate immune system of the nervous system, mediates neuroinflammation by the production of cytokines such as IL33, IL-8 and IL-1β. Microglial activation initiates inflammation of the neural tissues. The cytokines (IL-33) produced in the due process help in Aβ clearance whereas IL-8 and IL-1β cause synaptic dysfunction. This molecular mechanism reflects the complex.

Several hypotheses have been proposed to define the etiology of AD based on observed clinical, neuropathological features: cholinergic hypothesis, amyloid cascade hypothesis, and tau propagation hypothesis [25]. Some other potential hallmarks of Alzheimer’s dementia are mitochondrial dysfunction, calcium deregulation, neurovascular disintegration, neuroinflammation, metal ion dyshomeostasis, and defective lymphatic system [9,26,27,28]. However, the most well-known and defining features representing AD are Aβ accumulation, phosphorylated tau aggregation, and neuroinflammation [1,29,30]. In Figure 1, we have summarized the various AD hallmarks in the Alzheimer’s brain and have shown how excessive amyloid deposition leads to neuronal disease. As mentioned above, the amyloid cascade hypothesis postulates that APP metabolism and Aβ42 accumulation are the most important triggering factors for AD pathogenesis [31]. This hypothesis holds the accumulation of Aβ peptide responsible for the eventual loss of synapses and neuronal cell death [3,28]. An increasing body of evidence supports toxic Aβ as the primary cause of pathology, which can initiate neuronal dysfunction by inducing granulovacuolar degeneration, astrocytosis, microgliosis, and deficient endosomal transport when deposited extracellularly [32]. Moreover, the Aβ can also deposit around the small blood vessels of the brain, leading to the development of cerebral amyloid angiopathy (CAA), a common neuropathological condition usually occurring in AD patients, probably caused by the failure of Aβ clearance [33].

Tau hypothesis correlates AD pathology with the hyperphosphorylation and intracellular deposition of neurofibrillary tangles (NFTs) of microtubular protein tau [17]. It further elucidates that the propagation of the pathological form of tau protein from one neuron to another may drive the disease aggressively. Few studies linking both the above hypotheses highlight that aggregation of amyloid plaques leads to the activation of vaious kinases, causing hyperphosphorylation of the tau protein [18]. The deposition of plaques and NFTs initiate a neuroinflammatory response by activating microglia and astrocytes that detect aggregated proteins and promote the release of pro-inflammatory cytokines such as interleukin (IL)-1β, IL-6, and tumor necrosis factor (TNF)-α, as well as reactive oxygen species (ROS), giving rise to a chronic inflammatory process [34,35,36,37]. The link between Aβ and tau aggregation may be related to microglia activation. Indeed, it has been reported that soluble Aβ oligomers can activate microglial cells that in turn promote the hyperphosphorylation of tau with the subsequent formation of NFTs via cytokine release [38]. In addition to microglia and astrocytes, recent evidence has suggested that oligodendrocytes can also play a role in AD pathogenesis. Several cellular processes such as neuroinflammation and oxidative stress may trigger oligodendrocyte dysfunction and Aβ can impair the maturation of oligodendrocyte progenitor cells and the consequent formation of the myelin sheath [39]. Furthermore, the neuroinflammation and dysfunction of the blood–brain barrier (BBB) resulting from enhanced permeability and reduced expression of tight junction proteins due to increased production of Aβ, overexpression of matrix metalloproteinases (MMP)-2/-9, and ApoE, are also often independently linked with AD pathogenesis [40,41,42,43,44].

### 2.3. Diagnostic Biomarkers and Therapeutics

The definitive diagnosis of AD is only possible by post-mortem histopathological assessment of extracellular Aβ plaques and intraneuronal NFTs [45]. Although the treatment is mainly supportive with symptoms managed on an individual basis, some of the therapeutic options approved for AD from the FDA include the cholinesterase inhibitors such as donepezil, rivastigmine, galantamine (reversible AChEIs), and memantine (NMDA inhibitor) [14,46,47,48,49]. However, the effectiveness of these drugs is often questioned [50]. As the pathological changes silently occur in the brain over years before the onset of symptoms, the current challenge is the search for novel biomarkers for an easy and accurate diagnosis of the disease in its initial stages. The actual diagnostic methods rely on the measures of Aβ42, phosphorylated (p-tau), and total tau (t-tau) protein levels in the cerebrospinal fluid (CSF) of patients, in combination with advanced neuroimaging techniques such as magnetic resonance imaging (MRI) and positron emission tomography [51]. Different reliable biomarkers comprising several signaling proteins in blood plasma have also been discovered that can detect Alzheimer’s with approximately 90% accuracy even in patients with a mild cognitive impairment (MCI) which may later progress to AD [52]. A similar study with serum proteins, including a disintegrin and metalloprotease 10 (ADAM10), also retained diagnostic accuracy for the early diagnosis of AD [53]. Several blood-based microRNAs (Let-7b and microRNA-206) have also been found to have a strong correlation with cognitive decline and may be used as predictive biomarkers for AD [54]. Although promising, the use of these blood biomarkers in the clinical setting requires validation in further studies and standardization of pre-analytical sample processing and different methods.

## 3. Unsolved Mysteries in Alzheimer’s Disease Research

There are long-standing differences in opinion regarding the roles of soluble Aβ fibrils and tau tangles in ameliorating neurotoxicity, inflammation, and AD initiation. Due to the overrated role of amyloids in AD pathology, immunization against Aβ was presumed to be an effective strategy, which unfortunately failed to deliver expected outcomes in clinical trials [30,55]. In subsequent studies, failure to reverse AD pathology following Aβ42 targeting or delaying plaque formation led researchers to believe that Aβ42 deposition is not the sole reason for AD pathogenesis [56]. Nonetheless, this observation and other findings, such as genetic mutations in presenilin-1/-2 and abnormal APP processing in AD, emphasized a significant shift in the focus towards alternative theories [57,58].

The believers in tauopathy also have found challenges in establishing the correlation between the biochemical observations of tau tangles and the clinical progression of the disease in the patients [59]. The specific tau species involved in neurotoxicity are ambiguous and arduous to decipher from the results obtained in the macroscopic experimental setup [60]. Recent evidence indicates that it is not only the amyloid plaques but also the intermediate amyloidic species and oligomeric assemblies that are neurotoxic and may exaggerate the disease pathology [61,62]. The major drawback experienced in the current laboratory practices is that it is incapable of assessing these deleterious oligomeric assemblies due to the problems associated with its separation from the Aβ.

The absence of validated biomarkers, probably due to the inconsistent results produced due to analytical hindrances such as epitope masking and lack of reproducibility, prevents early detection of disease symptoms and poses additional challenges [63,64]. More robust investigation of genetic risk factors, the mechanism of receptor-mediated transport of Aβ and the role of interstitial fluid in regulating the metabolism of Aβ in vitro models need to be determined.

Activation of astrocytes leads to an exacerbated immune response causing neuronal damage and degeneration [65]. Contemporary experimental approaches involve mutant or transgenic animals with disease pathology leading to immense animal mortality [66].

Recently, exosomes have gained considerable attention both as a drug delivery system and a significant biomarker for diagnostics by offering prognostic information [67,68]. These small membrane-bound extracellular vesicles are ubiquitously released from eukaryotic cells to carry and deliver proteins, lipids, and nucleic acids, to the target cells [67]. Though most studies substantiate the benefits of exosomes in the clearance of proteotoxic burden by transferring neuroprotective substances between neural cells, recent findings revealed that exosomes are also involved in the transportation of protein aggregates involved in different neurodegenerative diseases [67,69]. Furthermore, these loaded moieties play a crucial role in AD pathology by spreading Aβ and hyperphosphorylated tau, inducing oxidative/proteotoxic stresses, neuroinflammation, and neuronal loss [68,69,70]. Since exosomes may prove to be a significant biomarker, better techniques are required to isolate exosomes at a large scale and perform experiments at a co-culture level. However, for the successful implication of these nanovesicles in the domain of exosomes, extensive research is required to ascertain the probable route of administration and safety aspects for clinical application.

## 4. Cellular and Animal Models of AD

### 4.1. In Vitro Models

The study of AD in vitro has been largely used to elucidate disease pathogenesis at molecular and cellular levels as well as for drug screening and discovery. Different cellular models have been developed to study various aspects of AD, including primary cultures, cancer cell lines, and induced pluripotent stem cells (iPSCs). However, cell culture systems cannot recapitulate the complex environment of the human brain and the interactions with other non-neuronal cells [71].

#### 4.1.1. Primary Cell Lines

Primary cell lines can be derived from transgenic animal or human patients. The major advantages in the use of these cultures rely on their easy availability and the potential to obtain different cell types, including specific neuron subtypes. Primary cultures have been extensively used to investigate the role of Aβ pathology both in astrocytes [72] and microglia [73]. Primary neurons, mainly derived from the hippocampus and cortex, were also employed to examine the neuroprotection mechanisms and the effect of Aβ oligomers on neuron function and apoptosis [74], as well as to reproduce the pathophysiological events occurring in AD, such as inflammation, altered signal pathways or epigenetic changes.

#### 4.1.2. Human Neuroblastoma (SH-SY5Y) Cell Lines

Originally isolated from human bone marrow with neuroblastoma, SH-SY5Y cells are derived from a neuronal lineage in its immature stage. According to the treatment, this cell line can differentiate into several various neural lineages which phenotypically resemble mature neuron-like features, including decreased proliferation rate, neuronal morphology, and expression of neuron-specific markers [75]. In regard to AD, SH-SY5Y cells can be modified to model some pathological aspects of the disease, such as neurodegeneration after exposure to Aβ oligomers [76], oxidative stress [77] and apoptosis [78], as well as to better understand the role of ApoE in AD [79]. Although this model has the potential to study the known molecular mechanisms associated with AD, it cannot fully recapitulate specific characteristics of the sporadic forms of the disease and age-dependent risk factors.

#### 4.1.3. iPSCs-Based Models of AD

Recent advances in iPSC technology have revolutionized the study of neurodegenerative disorders, given the limited access to living cells from brain patients. Reprogrammed from mature somatic cells of both familial (FAD) and sporadic AD (SAD) individuals, iPSCs can be differentiated into different disease-relevant cell types, maintaining the patient’s precise genome. The majority of studies performed on iPSC-derived neurons from fibroblasts of FAD and SAD patients showed high levels of Aβ42 and response to β- and γ-secretase inhibitors [80,81,82], as well as increased hyperphosphorylated tau, the two main pathological hallmarks of AD [81,83]. Regarding other cell types, iPSC-derived astrocytes from AD patients displayed severe pathology and dysfunction [84]. Additionally, iPSCs have also been used to investigate the role of the ApoE ε4 allele in different cell types, including neurons, astrocytes and microglia [85]. The inherent limitations of iPSC-derived two-dimensional (2D) cultures can be partially overcome by the generation of three-dimensional (3D) organoids, complex self-organized aggregates of different cell types derived from iPSCs that closely mimic the complexity of the brain’s architecture. Regarding AD, 3D cerebral organoids successfully recapitulate Aβ deposits, tau pathology and neuroinflammation [86,87].

### 4.2. In Vivo Models

In recent decades, different experimental models in various species have been generated to replicate AD pathology. Invertebrate animal models, including Caenorhabditis elegans, Danio rerio, or Drosophila melanogaster, have been selected for their short lifespan, well-characterized development and behavior [71]. However, mammalian models, especially mice, have been commonly used in AD research due to their similar anatomy to humans and easy manipulation [71].

#### 4.2.1. Transgenic Animal Models of AD

Since the discovery of AD-associated genes, different transgenic animal models have been generated by introducing the human mutant gene into the animal genome or by deleting a specific gene from the animal genome to develop the pathological hallmarks of AD. Many transgenic mouse models have been developed so far, harboring mutations in the APP, presenilin (PSEN1, PSEN2), MAPT genes or in combination (APP/Tau, APP/PSEN1 double transgenic mice, APP/Tau/PSEN1 triple transgenic mice (3xTg-AD) and five transgenic mice (5xFAD). However, these models do not reproduce all AD pathological features, as they mimic the genetic forms of AD without giving any information on sporadic AD. Single transgenic mouse overexpressing different mutations in APP gene and APP/PSEN1 double transgenic mice exhibited Aβ plaques and cognitive deficits but failed to develop NFTs, whereas the tau transgenic model showed NFTs, neuronal loss, and behavioral and motor impairments without developing Aβ plaques [88]. The two features of AD pathology were recapitulated with the generation of the APP/Tau double transgenic mice that displayed Aβ deposition, NFTs and motor deficits, representing a candidate tool to investigate the interaction between Aβ and tau protein. Compared to single and double transgenic models, the 3xTg-AD harboring mutations in APP, PSEN1 and MAPT genes exhibited more severe pathology but slow development of Aβ [89]. To accelerate the plaque formation, 5xFAD mice co-expressed five AD-linked mutations in human APP and PSEN1 genes, showing thus an early amyloid pathology, but lacking NFTs [90].

As ApoE represents the genetic risk factor for sporadic AD, transgenic, knock-in and knock-out mice expressing human APOE genes have been generated to investigate the mechanisms occurring in SAD. Knock-in mice expressing the human form of ApoE ε4 allele exhibited cognitive deficits [91] and high deposition of plaque or exacerbated tau-mediated neurodegeneration when crossed with APP or tau transgenic mice, respectively [92,93].

#### 4.2.2. Non-Transgenic Animal Models of AD

Non-transgenic animal models are used not only to study the classical AD hallmarks but also to model other pathological mechanisms, including oxidative stress, apoptosis, synaptic dysfunction, neuroinflammation, alterations in gut microbiota–brain axis, or autophagy [94]. As memory deficits and cognition loss are common traits of aged animals ranging from rodents to non-human primates, they can be used as a natural model of AD. Among them, the senescence-accelerated mouse-prone 8 (SAMP8) displayed age-related learning and memory decline as well as most features related to AD pathogenesis, such as oxidative stress, inflammation, Aβ plaques, NFTs, altered autophagy activity, and intestinal flora disruption, representing thus an ideal model to study this disorder [95]. Alternatively, animals can also be induced to develop AD by cerebral injection with Aβ synthetic peptide or other chemicals, by administering a high-fat diet to resemble metabolic abnormalities associated with AD, or generating radiofrequency lesions to the brain to induce cognitive deficiencies [88].

## 5. Microfluidics: An Overview and Biological Applications

**Figure 2:**
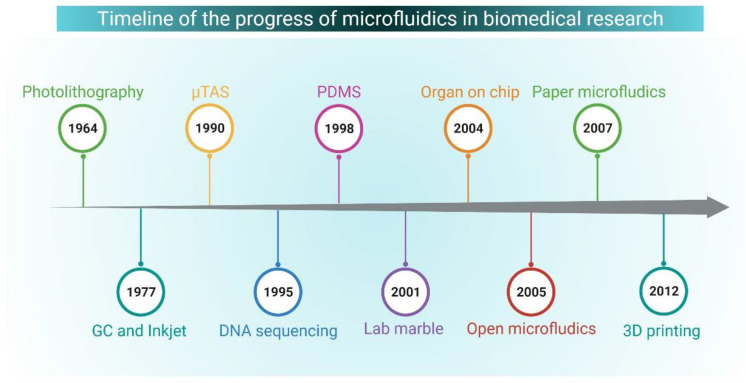
Timeline of the progress of microfluidics in biomedical research. Abbreviations: GC, gas chromatography; µTAS, micro total analysis system; PDMS, Polydimethylsiloxane.

The interdisciplinary field of microfluidics, derived from molecular biology, molecular analysis, and microelectronics, emerged in the late 1980s [96]. A timeline of the development of microfluidics from these physical and chemical innovations to its application in biological research has been provided in Figure 2. The emergence of this field began after discovering physical techniques such as photolithography and soft-lithography, later used for the fabrication of chips, and is still evolving with further technological advancements. The emergence of fabrication techniques facilitated the design and fabrication of chip-like 3D structures from solid substrates such as glass, silica, thermoplastics, etc. [96,97,98,99]. The first microfluidic devices or chips were made of silicon and glass. Still, due to their brittle nature, low gas permeability, and costly fabrication methods, they have never been considered an attractive option in microfluidics. Investigating alternative materials that could be optically transparent, easy to process, flexible, and comparatively cheap resulted in the discovery of several materials, which have been examined to date for the making of microfluidic devices (Table 1).

For the materials used in designing microfluidic devices, polydimethylsiloxane (PDMS), an elastomer introduced in the 1990s, is a material of choice for cell co-cultures [99,100,101,102]. As PDMS is compatible with cells, microfluidic devices made from it started to be used for cell biology applications and studies of co-cultures [98,103]. Technology is usually characterized as an engineering subject. Still, the implementation of the proof-of-concept experiments in the domain of microfluidics serves biologists and clinicians in enhancing capabilities in their everyday research. This technology allows the manipulation of small fluid volumes in a fabricated microscale system and has emerged as an excellent tool in modern biology. These microscale, multichambered tiny devices can grow cells and 3D tissues for biology research [20] and has enabled us to recreate experimental conditions at microscopic levels. This allows manipulation of biological specimens and cells at extraordinary spatiotemporal resolution and reveals otherwise hidden mechanistic insights, leading to a range of biological applications [104]. Properties such as rapid sample processing and precise control of fluids in microfluidic technologies have presented an attractive way to replace traditional experimental approaches. The microliter volumes of reagents mobile in laminar flow match with the biological microenvironments. Multiple diverse biochemical assays can be performed in a small volume, and the flow control feature at the micron level allows for improvement over the traditional macroscale assays. This is widely used in the imaging, bioinformatics, and molecular biology approaches [105,106]. Integration of fluid handling and signal detection features in microfluidics has allowed us to design cheaper yet sensitive point-of-care assay devices for different infectious diseases such as cancer, AIDS, malaria, SARS, dengue, etc. [107,108,109]. Even paper-based microfluidics such as DNA diagnostics have been developed in recent years, which are low-cost, multiplexed diagnostics [110].

Liquid marble (LM) is also a new type of digital microfluidics (DMF) that can be employed in a variety of biological applications. Cryoprotectant-free cryopreservation of mammalian cells using LM-based digital microfluidics is a potential method. This opens up new ways to cryopreserve rare biological samples without the risk of cryoprotectants causing toxicity [111]. LM can also be utilized for diagnostic testing, cell culture, and drug screening in the biomedical area [112]. DMF, a novel multifunctional microfluidics technology, offers a great deal of potential in the automation and miniaturization fields. In DMF, discrete droplets containing samples and reagents are controlled to implement a series of operations via electrowetting-on-dielectric. This process works by applying electrical potentials to an array of electrodes coated with a hydrophobic dielectric layer. DMF, unlike microchannels, allows for exact control of various reaction processes without the need for complicated pump, microvalve, and tubing networks. Other distinguishing characteristics of DMF include portability, lower sample consumption, faster chemical reaction time, versatility, and better integration with other technology types. DMF has been used in a wide range of fields (including chemistry, biology, medicine, and the environment) due to its distinct advantages [113]. Droplet-based microfluidics, which can be employed in drug discovery, transcriptomics, and high-throughput molecular genetics, has recently been reported. This enables researchers to work with relatively limited materials, such as primary cells, patient biopsies, or expensive reagents, and to perform tests at very high throughput (up to thousands of samples per second). The ability to undertake large-scale genotypic or phenotypic screens at the single-cell level is another advantage of the technology [114].

Isolated brain tissue, particularly brain slices, can be used to investigate neuronal function at the network, cellular, synaptic, and single channel levels. Neuroscientists have perfected ways for maintaining brain slice viability and function, settling on principles that are strikingly similar to the engineers’ approach to building microfluidic devices. With respect to brain slices, microfluidic technology may (1) provide better spatiotemporal control over solution flow/drug delivery to specific slice regions; (2) overcome the traditional limitations of conventional interface and submerged slice chambers and improve oxygen/nutrient penetration into slices; and (3) permit successful integration with modern optical and electrophysiological techniques [115]. Tissue culture (brain tissue slice) and drug screening have recently been performed using microfluidic platforms. In a study, microfluidic tissue culture system has been utilized to enable culturing of brain tissue slices for a sufficiently long period (up to 3 weeks) to facilitate studies on integration of neuronal stem cells into brain tissue and differentiation into dopaminergic neurons. This also allows for long-term culturing on a microscope stage for real-time microscopic imaging during neural stem cell integration experiments in brain slices [116]. This method has the potential to improve treatment success rates by identifying possible responders earlier in the treatment process and allowing direct drug testing on patient tissues during the early stages of drug development [117].

Cell-patterning techniques are also useful for neuron function and activity investigation and are one of the clear advantages of using microfluidics. So far, many neuron patterning techniques, such as in-mold patterning (iMP), and gel micropatterning by micro-casting, or by laser or protein patterning, have been reported. Many applications, ranging from neurodegenerative research to neural computation, require oriented neuronal networks with controlled connectivity. An efficient, directed, and long-lasting guidance of axons toward their target is required to establish such networks in vitro. However, the best guidance achieved so far relies on confining axons in enclosed micro-channels, making them difficult to investigate further. iMP improves axon confinement efficiency on the tracks by 10 to 100 times, resulting in mm-long, highly regular, and fully accessible on-chip axon arrays. iMP also enables well-defined axon guidance from small populations of multiple neurons confined at predefined places in μm-sized wells, thereby opening up new avenues for the construction of complex and precisely regulated neural networks [118]. Gel micropatterning by micro-casting is another neuron patterning approach. By using the repellency of agarose gel toward cell adhesion, patterned cultures of myoblasts and cortical neurons can be prepared. This technology is said to be beneficial for repellency-guided cell patterning in a variety of cell types, with applications in cell–cell interactions and axon guidance. With the repellency of agarose and no specific molecules for cell adherence, this technology is user-friendly and useful not just for micro-molding but also for cellular patterning [119]. Further, Stripe assays have been frequently used as in vitro test systems to investigate the responses of developing axons, as well as migrating cells, to established or novel guidance molecules. Silicon matrices are used to produce striped patterns of active molecules on a surface (referred as a “carpet”), which are then used to culture neurons or any other cell type. Purified proteins were utilized to produce stripe patterns on a silicon matrix [120].

**Table 1:** Properties of materials used in microfluidic chips.

Properties	Inorganic Materials	Elastomers	Thermoset	Thermoplastics	Hydrogel	Paper
**Examples**	Silicon/Glass	PDMS	Polyester	Polyethylene, Polystyrene Polycarbonate Polyurethane, Teflon, Cyclic Olefin Co-polymer (COC/COP)	Hyaluronic Acid, Agarose, PEG-DA, Alginate, PMMA, And Chitosan	-
**Biological Use**	Drug Screening, Assays	Assays, Cell Culture	Capillary	Electrophoresis, DNA Sequencing, PCR	Study Cell-Cell and Cell-Matrix Interaction	Diagnostics
**Young’s Modulus**	130–180/50–90	~0.0005	2.0–2.7	1.4–4.1	Low	0.0003–0.0025
**Fabrication Technique**	Photolithography	Casting, 3D Printing	Casting/ Photopolymerization	Thermomoulding	Casting/Photopolymerization	Photolithography, Printing
**Valving**	Yes	Yes	No	No	Yes	Yes
**Channel Dimension/Profile**	<100 nm/3D	<1 µm/3D	<100 nm/Arbitrary 3D	~100 nm/3D	~10 µm/3D	~200 µm/2D
**Thermostability**	Very High	Medium	High	Medium-High	Low	Medium
**Oxygen Permeability**	<0.01	~500	0.03-1	0.05–5	>1	>1
**Solvent Compatibility**	Very High	Low	High	Medium-High	Low	Medium
**Hydrophobicity**	Hydrophobic	Hydrophobic	Hydrophobic	Hydrophobic	Hydrophilic	Amphiphilic
**Surface Charge**	Very Stable	Stable	Stable	Stable	-	-
**Transparency**	No/High	High	High	Medium-High	Low-Medium	Low
**Cost**	High	Low	High	Low	Medium	Low
**Disadvantage**	High Cost, Brittle	Protein Adsorption, Permeability, Autofluorescence	Rigid, Poor Conductivity, Non-Recyclable	Low Melting Point, Brittle	Non-Adherent, Low Mechanical Strength	Porous, Sample Consumption
**Reference(s)**	[121,122]	[123,124]	[125]	[126,127,128]	[129,130]	[131]

Abbreviations: PEG-DA, Polyethylene Glycol Diacrylate.

**Figure 3:**
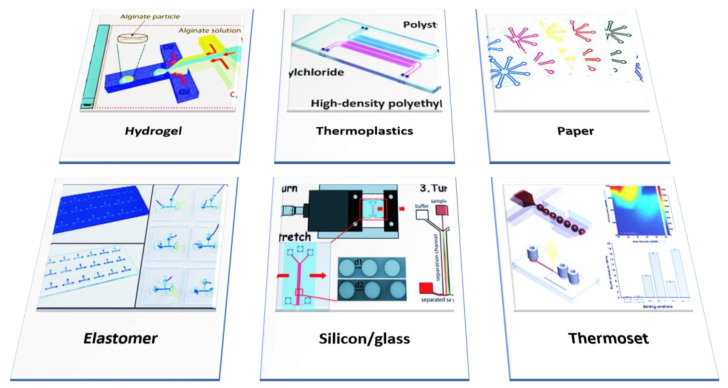
Schematic representation of the materials used for the fabrication of microfluidic chips. Hydrogels made up of natural materials, i.e., alginate, serve as matrices for culturing of cells in microfluidic chips. Thermoplastics such as polyvinyl chloride, polystyrene and high-density polyethylene are commonly used in fabrication. Moreover, the typical white color of paper makes it well suited for color-based detection methods in most assays and used for multiple bioassays in the form of origami-inspired folding devices. Elastomer is generally made up of PDMS. The glass-based microfluidic channel is made by the laser direct writing method. Thermoset, polyester-based, is a droplet-based device that can be used at different flow rates with three different oils.

Since the 2000s, organ-on-a-chiptechnology has been widely proposed and engineered on the structure and function of tissues and organs 2000 [132]. However, this has evolved rapidly in the past decade due to advancement in rapid prototyping methods such as 3D printing, widely used to produce 3D scaffolds for tissue engineering and devices mimicking a complex microfluidic environment [133]. The first “human-on-a-chip” cell culture systems to investigate physiological processes and “physiome-on-a-chip” systems to investigate novel compounds and their side effects on the human body have emerged [132,134,135,136]. The emulation of the pathophysiology of several neurodegenerative diseases in vitro through microfluidic devices has also risen rapidly [137,138]. A comprehensive study of the application of microfluidics in the study of neurodegeneration has been provided in the following sections. Several microfluidic tools available to date are shown in Figure 3.

## 6. Application of Microfluidics in Neurodegenerative Studies

Convergence of biology with engineering is evident in microfluidic devices used extensively nowadays in different domains of biomedical research contributing to a more powerful tool for drug delivery, point of care devices, and medical diagnostics [139]. Using microfluidics, a multichambered device can be readily prepared and used to grow neurites, glial cells, endothelial cells, and skeletal muscle cells, along with the maintenance of fluid isolation [140]. These devices can recapitulate organ-like structures and provide an opportunity to investigate organogenesis and disease etiology, accelerate drug discovery, screening, and toxicology studies by mimicking pathological conditions [141]. Utilizing hydrostatic pressure and chemical gradient profiles, localized areas of neurons grown in different compartments could be exposed to different kinds of insults applied insoluble form. A vast amount of literature exists highlighting applications of microfluidics in neurodegenerative diseases along with several neurodegenerative-disease-on-a-chip models focusing on AD, Parkinson’s disease, and amyotrophic lateral sclerosis [137,138,142,143,144,145]. Furthermore, the microfluidic system has been implicated in the study of regulated cell-cell interactions, elucidating the complexity of intercellular interactions in the neuroinflammation of growing primary brain cells.

It is well known that many brain cells interact with each other under varied conditions to cause neuroinflammation. The microfluidic devices facilitate cell culture, e.g., astrocytes in separate chambers exposed to varied situations. These chambers can be independently regulated and monitored for analyzing morphology, vitality, calcium dynamics, and electrophysiology parameters [146]. It has provided a platform to study neuronal cell death within the brain through simultaneous observation of neuronal connectivity and tau pathology [147]. Unlike 2D culture systems, these 3D cell cultures and microfluidic lab-on-a-chip technologies with in vitro microfluidics systems do not lack the mobility of the cultured cells allowing a better physiological extracellular environment, for examining, neuron-glia interactions minimizing animal morbidity and mortality [148,149,150]. With the help of 3D culture techniques, the discrepancies in the results of in vitro culture systems and animal models in drug discovery can be avoided [151].

Studying brain development and degeneration at the cellular level suffers several limitations due to the inability to isolate cell culture systems, the absence of an organized physiological neuron connection architecture, and so forth. In this regard, microfluidic systems present an irreplaceable tool to simulate the BBB microenvironment, study axonal functions’ construction of neuronal networks, and develop drug delivery systems through devices such as axonal diodes and minimized wireless devices [22,145,152,153,154,155]. Furthermore, the technology has led to the minimization of animal models in the study of neurodegenerative diseases, drastically cutting down labor-intensive efforts, time, and animal mortality. Besides, the discrepancies that arise due to species differences between humans and animal models can also be minimized.

The lab-on-chip technologies, with features on a similar physical scale to that of cells, have facilitated the study of complex neural signaling pathways to detect abnormalities, and check whether the application of inhibitors can reverse these without the requirement of animals [156,157]. The microfluidic entities can replicate complicated cell biological processes that control synaptic function, visualize them and manipulate synaptic regions and presynaptic and postsynaptic compartments independently under in vitro conditions, and manipulate synapses and presynaptic and postsynaptic cell bodies independently [101]. Studies show that synapses lose native circuitry and order due to the dissociating of neurons for in vitro studies. The organization of cultured neurons and their connections can be improved and restored by mimicking the natural circuitry in vivo conditions through microfluidic approaches [101]. With the help of microfluidic culture devices, two distinct micro-environments can be established, which may be maintained in fluidic isolation to allow for targeted investigation and treatment.

A compartmented kind of setup to co-culture a wide variety of cells is required to understand the mechanisms of a range of neurodegenerative diseases and model neuromuscular signaling [158,159]. The microfluidic devices fulfill all these requirements and mimic the unique anatomical and cellular interactions of this circuit [159,160]. 3D assay systems have been developed, human brain models allowing the measurement of action potential and velocity, monitoring cell growth, drug discovery, and study of neural–glial interactions and various neurotrophic factors [156,161]. Furthermore, microfluidic neuromuscular co-culture enables innervation by axons crossing from the neuronal to the muscle compartment [162]. The same setup can be used to decipher the impact of genetic alterations on the synaptic function of CNS disorders [163]. Therefore, microfluidics applied widely in various studies of disease, including neurodegeneration. Similarly, its impact on the research and development of AD is overwhelming and promising.

## 7. Impact of Microfluidic Tools in Alzheimer’s Disease Research: Recent Developments

Advancements in microfluidic technology have played a significant role in accelerating the research dedicated to the field of AD, as with other diseases, in terms of both drug discovery, exploring novel drug targets, understanding the pathophysiology, or discovering novel biomarker-based diagnostics. A list of such initiatives has been provided in Table 2. Novel AD models, which are more helpful in mimicking the complex features of AD pathology, have started to replace the traditional models. The 3D culture platforms are more suitable for studying AD pathophysiological mechanisms involving cell–cell interactions, controlled flow dynamics, circulating blood cells, and a brain-specific microenvironment. In a study, distinct roles of Aβ on microglial accumulation have been elucidated by quantifying microglial responses in order to gain insights into the pathophysiological role of microglial migration [164].

Similarly, the effects of axonal trauma on the neuronal networks of primary brain cells and the role of astrocytes were studied on a microfluidic platform [165,166]. The ease, accuracy, and reproducibility of the experiments encouraged a more significant number of studies. Apart from basic research, many disposable biosensors for early detection of AD biomarker ADAM10 and Aβ peptide in the serum have also been developed (limit of detection ~0.35 fg/mL) [167,168]. These low-cost diagnostic kits exhibit better accuracy and sensitivity than the well-established enzyme-linked immunosorbent assay test.

The emerging role of exosomes in the detection and study of AD has created the need for large-scale separation of exosomes, which is cumbersome and challenging with traditional techniques like ultra-centrifugation. Microfluidic devices are emerging as an ideal tool for exosome separation and have also started to gain recognition as excellent exosome detection tools [169]. These miniaturized platforms enable quick and cheap processing of nanovesicles even in the small volumes of liquid samples. Several microfluidic chips based on 3D neuro spheroids have been developed to mimic in vivo brain microenvironment [143]. These kinds of 3D culture-based microfluidic chip provide in vivo microenvironments for high-throughput drug screening and allow the investigation of dendrite-to-nucleus signaling [170]. Synthetic models with AD features such as aggregation of Aβ, and accumulation of phosphorylated tau protein with neuroinflammatory activities have been produced to emulate pathological states. A triculture in vitro model comprising the combination of neurons, astrocytes, and microglia has evolved to address the physiological features and study the durotactic behavior of cells [171]. The human AD triculture model provides an opportunity to learn about microglial recruitment, neurotoxic activities, and astrocytes [171]. A co-culture system with segregated cell bodies, while simultaneously forming myelin sheaths, could also be obtained through a microfluidics approach [172].

These studies claim to reverse the demyelination of axons which can recover the loss of sensory and motor function with the help of co-cultures. The microfluidic devices allow the study of AD-derived tau propagation from neuron to neuron. Application of microfluidic cell culture must be undergone only upon testing the cell lines with the PDMS formulations, checking for leaching of toxic compounds, and examining that the medium composition is well adjusted to suit the device and cells. Microfluidic systems present a reliable method to mimic in vivo fluid conditions of neural tissues by generating gradients to allow the diffusion of two separate fluid phases at the interface [36]. The microfluidic technology facilitates understanding of the mechanism of Aβ under interstitial fluid flow conditions. These kinds of 3D culture-based microfluidic chips provide in vivo microenvironments for high-throughput drug screening [106,132]. These devices have also been used to isolate axons and the cell body to study the targets of excitotoxicity observed in neurodegeneration. In another study, the distal axon is the main target. These models can be widely used for basic mechanistic studies involved in the interaction between neural-glial cells and drug discovery. The microfluidic approach has also been used to grow a 3D human neural cell culture wherein a BBB-like phenotype was developed. The generation of such a phenotype helps in screening novel drugs capable of passing through the BBB to reach deeper neural tissues [148]. This technology facilitates the culturing of cortical neurons in two distinct cell compartments of the same microfluidic device to generate neuronal networks [173]. This setup can bring axonal degeneration in the distal axon chamber without degenerative changes in the untreated somal section [174]. Insults to the selective areas of neurons can be obtained without affecting other neurons by applying hydrostatic pressure [142].

**Table 2:** Details of microfluidic devices and their application in the AD research.

Cells/Peptide	Flow Control Device	Flow Surface	Active/Passive	Application	Reference(s)
Axon	NA	Glass	P	Study axonal function	[154]
Neural Progenitor Cell	Osmotic micropump	-	A	Study the neurotoxicity of amyloid beta	[36]
Neuron	Osmotic micropump	Glass	A & P	in vitro brain model, high-throughput drug screening	[143]
Brain Cells	Pneumatically-driven pumps	Polysulfone	P	To provide MPSs for in vitro drug discovery	[175]
Aβ42 Peptide	Precision pump	Glass	A	Aβ (1–42) detection	[168]
Aβ Peptide	Syringe	-	A	-	[176]
Axons	N/A	Glass	P	Study impaired axonal deficit	[156]
Axons	N/A	MEA	P	Investigate axonal signals in developmental stage	[177]
Neurites	Syringe	Glass	A	Study durotactic behavior of cells and neurite growth	[161]
Axons	Gravity/Hydrostatic pressure	PCB/Glass	P	Study axonal physiology and modeling CNS injury	[178]
Soma and Axon	N/A	Glass	P	Compartmentalizing the network structure into interconnected sub-populations	[179]
Hippocampal Neuronal/Glia Cells	Pressure gradient	Glass	P	Probing the functional synaptic connectivity between mixed primary hippocampal co-cultures	[163]
Dendrite	N/A	PDMS	NM	Investigate dendrite-to-nucleus signaling	[170]
Oligodendrocyte	N/A	Glass	P	-	[172]
Drg/Mc3t3-E1	N/A	Glass	NM	Mimicking the in vivo scenario to study the interaction between the peripheral nervous system and bone cells	[160]
Nmj	Pipette	Glass	N/A	Study subcellular microenvironments, NMJ formation, maintenance, and disruption	[162]
Axons	Pipette	Glass	P	Perform drug screening assays	[180]
Dendrites and Somata	Syringe	Glass	A	Manipulate synaptic regions and presynaptic and postsynaptic compartments in vitro	[101]
Glial Cells/Motor Neurons	N/A	Glass	P	Study interactions with glial cells and other skeletal cells in the chamber	[159]
Astrocyte	N/A	acrylic plate	P	AD triculture model showing beta-amyloid aggregation, phosphorylated tau accumulation, and neuroinflammatory activity	[144]
Tau	N/A	Glass	P	Study effects of tau on mitochondrial transport	[181]
(Aβ) Peptides	N/A	Glass	P	Study effects of local Aβ stress on neuronal sub-compartments and networks	[182]
ADAM10	Syringe	N/A	A	ADAM10 biomarker detection in plasma and cerebrospinal fluid	[167]
Tau	N/A	Glass	P	Quantify AD-derived Tau propagation	[147]
Aβ	N/A	Glass	P	Study roles of Aβ on microglial accumulation	[183]
Aβ	Syringe	Overflow microfluidic networks	A	Study cell-to-cell communication, role of astrocytes derived from cortex and hippocampus on neuronal viability	[146]
Axons	-	Glass	-	Study mechanisms of indirect axonal excitotoxicity	[174]
Neurites	Hydrostatic pressure	Glass and Polystyrene	P	Grow neuronal culture	[142]
Cortical Neurons	Pressure difference	Glass	P	Synthesize experimental models emulating pathological states	[173]
Ren-WT/Ren-AD Cells	N/A	Glass	P	Grow 3D human neural cell culture, screen novel drugs capable of passing through the BBB to reach deeper neural tissues	[148]
Protein	N/A	Glass	P	Detect protein aggregation	[184]
Axons	Hydrostatic pressure	Glass or Polystyrene	P	Study localized axon-glia interaction and signaling	[185]
Axons	N/A	Glass	P	Examine axonal trauma in neuronal networks	[166]
Axons-glia	Hydrostatic pressure	Glass	P	Study axon-glia interactions	[186]
Neurites	Syringe	Glass	A	Investigating chemotaxis of neutrophils	[187]

Abbreviations: MPSs, Micro-physiological systems; DRG, Dorsal root ganglion; NMJ, Neuromuscular junction; MEA, Microelectrode arrays.

## 8. Challenges in the Application of Microfluidics in the Alzheimer’s Disease Research

Although microfluidics provides a state-of-the-art facility that enables investigations in biomedical research, there are many challenges that need to be addressed before the optimal utilization of this field’s potential. Experts believe that the area of microfluidics research needs to grow further in order to outperform existing laboratory methods and overcome barriers that hinder researchers from adopting microfluidic-based devices as a common research tool.

First of all, the lack of precise fluid handling techniques at such a microscopic level poses great difficulty in attaining the exact quantity of reagents for performing molecular experiments. Though achieved once, it becomes difficult to replicate the experiments with acceptable accuracy. The second major problem is that it is difficult to scale up the experiments under the same experimental conditions with the same volume of reagents. This is because of inability in fluid handling and duplicating culture or reaction conditions. Often cells may respond differently toa change in the substrate of microfluidic devices. Thirdly, the majority of the culture protocols have been optimized on polystyrene culture plates, a significant component in macroscale devices, unlike microfluidic cell culture devices that use PDMS. New production techniques favoring mass production such as microfluidic hot embossing in polystyrene have been found useful in minimizing the risk of translation failure in microfluidic devices, yet PDMS is the most commonly used substrate for fabricating these devices [188].

Any variation in the reagent volume or reaction conditions leads to inaccurate results and protocols. Moreover, a direct comparison with the macroscale experiments become very difficult as a change in the substrate may hinder the transition of the protocols to the microscale levels. Studies indicate that PDMS may absorb or adsorb the biomolecules from the medium, causing biased experimental conditions [189,190]. Absorption and/or adsorption of reagents will alter the reaction volumes, which is another demerit that microfluidic devices currently face. In addition, we do not know whether PDMS, a material known for its transparency and gas permeability, has any impact on cellular behavior. Since it is the material of choice at present, ascertaining its effect on cellular behavior is essential.

Excessive permeability, technical robustness, and other properties might lead to sample drying and change in osmolarity, posing a considerable obstruction. Samples collected on chips/microfluidic channels for analysis using chip-based PCR, histochemistry, western blots, or MS-Spectrometry will fail to give accurate results upon a slight change in the volume of reaction constituents [104,191]. Additionally, these experiments require the reagents to be properly mixed, but microfluidics produces slow diffusive mixing due to laminar flows, posing a major limitation for these systems wherein fast homogenization is required [192].

The lack of a universal blood substitute or standard culture media that supports all types of tissue is an additional setback. Other drawbacks that must be addressed in the future for the optimal application of microfluidics in Alzheimer’s research is its interdisciplinary nature, wherein standardized protocols are generally absent. A combined effort of engineers and molecular biologists is required to fabricate new device designs and carry out biologically relevant experiments [36]. As a range of cell lines are cultured in these devices, generalization in device designs is difficult.

It is well known that physical parameters such as flow, pressure, temperature, pH, and real-time monitoring are equally important in carrying out biological experiments with accuracy. To ascertain these parameters, newly designed chips are now well integrated with the in-line sensors and microfluorimetric imaging facilities, but the chip still lacks features such as feedback control, continuous monitoring, and experimental sample processing. Unlike macroscopic laboratory practices, an automated control system is required to expand the domain of users and replace the 2D or 3D culture systems. The 3D tri culture AD model is gaining popularity as it is undoubtedly advanced over in vitro human AD models. Nonetheless, physiologically relevant in vivo studies are still required to confirm its clinical utility [144].

## 9. Conclusions

Even after a century of extensive research, the field of AD requires more work in the appropriate direction to come up with effective diagnostics and therapeutic cures [12,56,193]. The crucial research problems are challenging with current macroscopic laboratory equipment and practices. The research is at a crossroads where rigor is required to determine the right direction and appropriate focus. Microfluidic systems facilitate work on functional organs at the level of molecular analysis, significantly minimizing the complications involved in handling in vivo systems. These devices outperform age-old methodologies through features such as rapid sample processing, fluid control, flexibility of design, controlled co-culture, reduced reagent consumption, low contamination risk, and efficient high throughput experimentation. Undoubtedly, these novel neurotechnological tools are very useful in gaining an in-depth understanding of the brain’s functions and discovering novel therapeutic strategies for neurological disorders like AD. However, the extent to which this technology can serve in AD detection and management is still in a nascent phase. This is because the technology has not been developed to recapitulate biological responses to various stimuli such as chemicals or toxins. Although organs-on-chips may lead to the identification of biomarkers and validation of lead drug candidates, clinically relevant PK/PD models are required to determine the drug doses. In this regard, better scaling approaches to keep an account of fluid flows and volumes of distribution would ensure functional PK/PD models. It is doubtful that organs-on-chips will replace animal testing anytime soon, as the organ function and regulatory requirements are highly complex. Nonetheless, these low-cost techniques are up-and-coming and have accelerated the pace of AD research.

## Data Availability

Not Applicable.

## Abbreviations

AD	Alzheimer’s disease
ADAM10	A Disintegrin and Metalloprotease 10
Apo E	Apolipoprotein E
Aβ	Amyloid-beta
CD33	Complementary determinant 33
IL	Interleukin
MMP	Matrix metallopeptidase 9
NMJ	Neuromuscular junction
PCR	Polymerase chain reaction
PDMS	Polydimethylsiloxane
PK/PD	Pharmacokinetic/Pharmacodynamic
TNF-α	Tumor necrosis factor-alpha
TREM2	Triggering receptor expressed on myeloid cells2
